# Meaning and medication: a thematic analysis of depressed adolescents’ views and experiences of SSRI antidepressants alongside psychological therapies

**DOI:** 10.1186/s12888-018-1961-y

**Published:** 2018-11-28

**Authors:** Rita A. Maroun, Lisa A. Thackeray, Nick Midgley

**Affiliations:** 1 Faculty of Brain and Language Sciences, University College London, London, UK; 2 Anna Freud National Centre for Children and Families (AFNCCF), London, UK; 3 The Child Attachment and Psychological Therapies Research Unit (ChAPTRe), University College London and Anna Freud National Centre for Children and Families, London, UK

**Keywords:** Depression, Antidepressants, SSRIs, Adolescents, Patient perspectives, Medication use, Qualitative

## Abstract

**Background:**

Adolescence is a key period of risk for the emergence of Major Depressive Disorder (MDD). The prescription of selective serotonin re-uptake inhibitors (SSRIs) for the treatment of depression in adolescents is an issue of worldwide controversy, and evidence regarding their safety and efficacy is inconclusive. In the UK, NICE guidelines have recently recommended offering SSRIs to adolescents alongside psychological therapy or on their own if therapy is refused. Thus, SSRIs are increasingly becoming a major component of treatment for adolescents. This study qualitatively explored adolescents’ views and experiences of SSRIs within their accounts of engaging in a psychological therapy for depression, particularly focusing on meanings they attached to medication-use.

**Methods:**

The qualitative study reports data from semi-structured interviews conducted 12-months post-treatment with 12 adolescents who were clinically referred and treated for depression as part of the IMPACT trial. The interviews were analysed using Thematic Analysis.

**Results:**

Four themes were identified: ‘a perceived threat to autonomy’, ‘a sign of severity’, ‘a support, not a solution’, and ‘an ongoing process of trial and error’.

**Conclusions:**

This study highlights the value of bringing adolescents’ voices into the broader debate on the use of antidepressants in their age group and in the development of future guidelines. Future implications for research and clinical practice are discussed.

## Background

The safety and efficacy of antidepressants for adolescents is an issue of ongoing worldwide controversy due to concerns about elevated risk of self-harm and suicidal behavior [1, 2]. For over a decade, numerous studies have examined the safety and efficacy of Selective Serotonin Re-Uptake Inhibitors (SSRIs) in adolescents, but results have been conflicting [see 1]. A recent systematic review and meta-analysis of clinical trials for several SSRIs found that serious risks, including suicide and aggression, were underreported and the extent of potential harm is not always discernable [3]. Still, prescription of antidepressants for under 19’s increased worldwide from 2005 to 2012 [4]. In the UK, first-ever prescriptions of antidepressants for 3–17-year-olds approximately doubled between 2006 and 2015 [5].

The National Institute for Health Care and Excellence (NICE) makes recommendations for treatment in the UK based on systematic reviews of best available evidence [6]. For adolescents presenting with moderate-to-severe depression, previous guidelines recommended an evidence-based psychological therapy as first line treatment and cautioned against prescribing antidepressants unless the adolescent is unresponsive to psychological therapy, in which case they recommended Fluoxetine, a Selective-Serotonin Re-Uptake Inhibitor (SSRI), in addition to psychological therapy [6]. The 2015 amendment recommended offering combined psychological therapy and Fluoxetine as an alternative first line of treatment. SSRIs can also be offered on their own if psychological therapies are declined, albeit with close monitoring. These amendments could mean that more adolescents in the UK will be offered antidepressants and potentially at an earlier stage of treatment. A recent study suggests that SSRIs are being prescribed in the absence of psychological therapies, contrary to NICE recommendations; at least 19% of 465 adolescents were prescribed SSRIs prior to beginning psychological treatment [7].

The rising prescribing trends, aforementioned prescribing practices, and the amendment in guidance all suggest that antidepressants are increasingly becoming a major component of treatment for adolescents. Further, the inconclusive evidence regarding safety and efficacy of antidepressants highlights the need to better understand the impact of these medications on adolescents. Developing an understanding of the impact of antidepressants that is grounded in adolescents’ perspectives and narratives can offer new insight into current prescribing practices and the broader debate around antidepressant use, especially given that they are the main stakeholders in treatment.

Antidepressant use in adolescents has been extensively researched (e.g.[1, 8, 9]). However, only a small number of studies have focused on adolescents’ own understanding and interpretation of antidepressants as well as their subjective experience of the effects [10–13]. The available literature on antidepressants, and psychiatric medication more broadly, suggests that adolescents attribute different meanings to medication, which could coincide with developmental issues pertinent to adolescence, such as identity formation, autonomy, and social acceptance [10–16]. For instance, adolescents in one study describe hiding their medication use due to feelings of shame and differentiation from peers, stating that it marked them as ‘defective’ [15]. In other studies, adolescents have reported that they view medication use as contradictory to their identity as ‘normal’ and ‘autonomous’ teenagers, and that this can act as a barrier to accepting and adhering to treatment [10, 12, 15]. One study found that adolescents felt resistant to even seeking help for depression due to fear of being prescribed medication [10].

Together, these studies highlight the importance of exploring personal meanings that adolescents may attribute to antidepressant medication throughout the treatment process, and how these meanings impact not only their acceptance and adherence to the treatment, but also their developing sense of self. Further, for adolescents who choose to take it, medication is a daily and possibly important part of the experience of depression and treatment. This study therefore aimed to qualitatively explore adolescents’ views and experiences of SSRI medication within in their accounts of overcoming depression; specifically, to identify patterns of meaning that these adolescents attribute to medication within their broader experience of depression and treatment.

## Methods

### Setting

This study drew on interviews with adolescents who participated in Improving Moods with Psychoanalytic and Cognitive Therapies (IMPACT), a randomized controlled superiority trial which compared the effectiveness of three psychological therapies for adolescent depression (see [17, 18]). The study recruited 470 adolescents aged 11–17 years with a diagnosis of Major Depressive Disorder (MDD) from 15 Child and Adolescent Mental Health Services (CAMHS) across three UK regions. Adolescents were randomly allocated to one of three manualized treatments: Cognitive Behavioral Therapy (CBT), Short Term Psychodynamic Psychotherapy (STPP), and Brief Psychosocial Intervention (BPI). Adolescents were offered SSRIs during and post-treatment in combination with the allocated therapy as recommended by NICE guidelines. IMPACT-My Experience (IMPACT-ME), a qualitative, longitudinal study, took place alongside IMPACT. Interviews were conducted with adolescents participating in the trial before therapy (baseline), at the end of therapy, and one year after (follow-up) see [19].

### Data collection and sampling

The current study drew on follow-up data from the ‘Thinking back about therapy’ interview, a semi-structured schedule which invites adolescents to reflect on their experience of overcoming depression soon after therapy ended. Interviews were carried out by research psychologists with additional training in in-depth interviewing and lasted between 30 and 90 min. However, medication was not the primary focus of the interview. Therefore, any mention of medication was either in response to a specific question related to medication, ‘was medication ever discussed with you?’ or volunteered by the adolescent.

Interviews with all 70 adolescents who took part in the IMPACT-ME study were first reviewed. As per the current study’s aim to identify patterns of meaning that are attributed to medication, adolescents who gave a yes / no answer to the interviewer’s question but did not expand the discussion beyond a single statement or did not elaborate beyond logistical information (e.g. when medication was suggested, which medication they took, dosages) were excluded. This resulted in a sample of 12 interviews in which adolescents spoke about their views and experiences of being offered medication and/or of taking medication. Given that this is a relatively under-explored topic, the accounts of both adolescents who had and had not taken medication were included in the sample.

### Participants

The 12 adolescents (10 female, 2 male) were 13–18 years old at baseline. All sought and received treatment as part of IMPACT and met diagnostic criteria for moderate-to-severe unipolar depression at time of recruitment, as assessed by Kiddie-SADs [20]. Exclusion criteria were generalized learning difficulties, pregnancy, primary diagnosis of bipolar Type 1, schizophrenia, pervasive developmental disorder, and eating disorders. Two had received treatment in the STPP arm, five in the BPI arm, and five in the CBT arm. Three adolescents expressed their views about medication despite not having been offered it, three had refused to take medication when offered, and six had taken medication – four of whom had stopped. Of the adolescents who took medication, three of the adolescents had been prescribed prior to being recruited and receiving psychological treatment in the study. When examining their baseline characteristics, no differences were identified between this sub-group and the complete set of participants in the IMPACT-ME study, other than a slightly lower proportion of young people in the STPP arm of the study.

### Study design

The current study employed a qualitative design to explore the sections of the interviews in which adolescents discussed medication with the aim of obtaining an in-depth, nuanced account, emphasizing adolescents’ personal views and/or experiences of medication whilst also drawing broader patterns of meaning across interviews. To facilitate this, interviews were analyzed using thematic analysis (TA), a flexible method of qualitative analysis that allows researchers to actively identify, analyze, and report patterns of meaning in data, while providing a detailed and rich account of themes [21]. Following Braune and Clarke’s [21] six-phase approach to TA, ‘familiarization’ with the data was achieved by listening to audio-recordings and reading entire transcripts to grasp contextual details of adolescents’ broader narratives, noting initial ideas about the data. In phase two, the first author coded areas that discussed medication by systematically identifying and highlighting interesting features and labelling them according to their content. Each extract was coded with as many codes as possible until a list of codes had been generated. In phase three, the author identified patterns in the codes across the whole dataset and grouped them into potential themes via mind-map, whereby different combinations of codes were arranged and re-arranged until candidate themes and their sub-themes had been devised. Phase four involved refining identified themes in a recursive process; coded data extracts were tabulated under themes and were then read together under each theme to determine whether they adequately and coherently captured data patterns and to ascertain whether themes were sufficiently supported. Afterwards, the author re-read the interviews to determine whether themes reflected the data and captured an overall picture of meanings adolescents attributed to medication. At each stage the first author discussed and refined overarching themes and sub-themes with the co-authors. Extracts were re-coded again and any data missed in previous phases were added under identified themes. In phase five, a detailed analysis identified the over-arching message for each individual theme in relation to research aims, with attention to nuances while still highlighting broader ‘story’ that the combined themes tell about the data. Finally, each theme was named and given a concise definition describing its content.

### Trustworthiness and credibility of the analysis

Braune and Clarke’s [21] 15-point-criteria for conducting ‘good’ thematic analysis were followed, with a particular emphasis on trustworthiness, credibility, transferability, dependability, and confirmability [22]. Throughout the process, the research team took a position of reflexivity [23], continuously acknowledging and evaluating the impact of their own perceptions, experiences, interests, and background. Interpretations of data were discussed with co-authors and examined by peers and necessary changes were made. Further, the data was iteratively coded three times. A journal was used to document and review thought progression, decisions, and discussions. An audit trail documented how data was coded and grouped into themes. The research team attempted to ‘stay close to the data’ during analysis and used direct quotes from adolescents when reporting findings to allow readers to check the analysis against original accounts. Additionally, information about the study setting and adolescents’ individual contexts were incorporated into write-up to aid transferability.

## Results

**Fig. 1:**
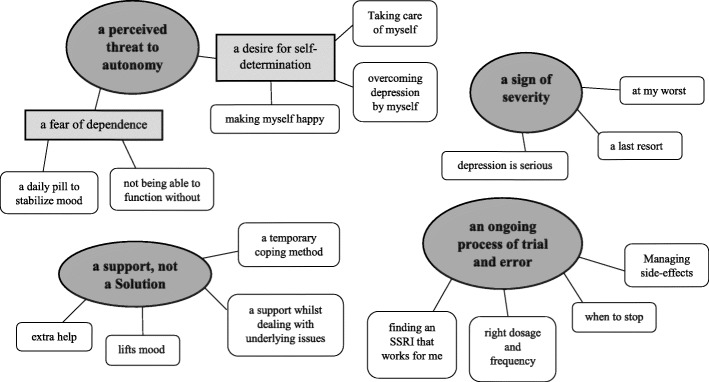
Final thematic map: themes, sub-themes, and features

Four interrelated themes were identified in relation to the study aim. Three of these themes were central across the entire dataset: ‘a perceived threat to autonomy’, ‘a sign of severity’, ‘a support, not a solution’. One theme was specifically salient to adolescents who had taken medication: ‘an ongoing process of trial and error’. These themes are reported along with their respective sub-themes (see Fig. 1).

### Theme 1: ‘A perceived threat to autonomy’

For some adolescents, medication seemed to be a threat to their sense of autonomy. Taking medication appeared to challenge their view of themselves as independent and free from external control. This theme was characterized by two interconnected sub-themes: ‘a fear of dependency’ and ‘a desire for self-determination’. These adolescents either never sought medication, refused to take it when offered, or took it and stopped. For all these adolescents, this perception had implications for whether they took medication as well as adhered to it.

#### ‘A fear of dependency’

A fear of dependency was present in many adolescents’ narratives—whereby taking medication seemed to demarcate a forfeit of their independent functioning to an ‘added chemical’ in order to regulate mood and manage depression.

Adolescents spoke about how taking a daily pill to manage their depression can be daunting. Talia, who took medication, described how relying on medication every day to keep her mood stabilized was simultaneously a source of anxiety and confusion:

*“It’s annoying I have to take these pills every day…if I forget it could be bad(...)It’s unnerving when you’re feeling happy and having a side thought going yeah but are you happy or is it just added chemicals making you feel happy?” - (Talia)*

For Talia, taking medication led her to question her ability to function without it and interfered with her perception of her emotions as being her own. She recalled her decision to stop taking medication because she felt it was a risk worth taking in exchange for being free from having to rely on it daily, even if this meant ending up in hospital:

*“It wouldn’t be worth having to take pills every single day just to stop that one day coming around every now and again(...)so I was like I might as well stop, see what happens(...)worst comes to worse I might end up in hospital again” - (Talia)*

Adolescents also described a fear of being physically dependent on anti-depressants. For example, for Mathilda, this seemed to be associated with a fear of experiencing a difficult withdrawal, making her hesitant to stop taking medication:

“*I didn’t wanna depend on it too much …but at the same time I was worried about coming off it, especially doing A-levels cos it’s a stressful time” - (Mathilda)*

Whereas, for Elizabeth, these fears, which arose from family experience, contributed to her decision not to take medication:

*“My Mum’s been on the medications and I don’t really wanna go on it cause I’ve seen her try and come off it. It’s taken her three or four attempts before she could come off it, so I said ‘I don’t wanna be dependent on it’.” - (Elizabeth)*

#### ‘A desire for self-determination’

This sub-theme delineates a simultaneous desire to manage depression via one’s own will, whereby change has to be self-induced and on one’s own terms.

Many adolescents who talked about their fear of dependency also expressed a desire to overcome their depression by their own will and self-determination: *“I want to get over this myself”* (Elizabeth). Talia, who took medication, spoke about wanting to be free from depending on it to manage her mood and wanting to regain her sense of self-determination:

*“I’m capable of making myself happy(...)so why am I having to take extra chemicals in my body that can cause these side effects when I could just work on myself and do it that way?(...) like I need to stop this. I was getting to a point where it’s like I can kind of take care of myself.” - (Talia)*

Talia describes medication as providing a lift in mood at the price of enduring unwanted side effects, thus impinging on a sense of bodily control in an undesirable way. For Talia, this seemed to elicit a desire to regain a sense of control over her body, which, together with her belief that she can make herself happy, contributed to her decision to stop medication. For Callum, his preference to overcome his depression by his own means led to his decision not to take medication at all:

*“I wouldn’t even wanna take medication. I’d rather just deal with it my way.” - (Callum).*

Overall, the need for medication is understood here as a sign of an inability to cope with depression via self-determination. Further, adolescents seemed to have a shared perception that taking medication would take away a degree of control over their bodies and impinge on their ability to take care of themselves.

### Theme 2: ‘A sign of severity’

Almost all adolescents identified a particular point at which they deemed or would deem medication to be necessary, referring to how low they perceived their mood to be and its impact on their functioning.

Medication was seen as necessary when depression was experienced as particularly severe. The adolescents who had never considered or had declined taking medication spoke of how they were not at a point in their depression that warranted its use, “*I don’t think my depression was that serious, so I didn’t take medication”* (Priya). Further, medication seemed to be reserved for a level of severity that adolescents were able to recognize:

*“I think that should be the last resort, I don’t think you should put everyone on meds because sometimes it’s not serious for them to be on meds. For me, it was not that serious” - (Natasha)*

Similarly, adolescents who had taken medication referred to a certain point at which they felt their mood was so low that they decided its use was necessary for them to be able to manage. For example, Steven described feeling so overwhelmed and trapped by his depression that medication was thought to be a necessary next step:

*“I got prescribed antidepressants because I felt really down…like this constant feeling of down(...)I’m depressed in a way that makes me feel like I can’t do anything about it.” - (Steven)*

Lana, who declined medication, recalls that she considered taking it when her depression was so severe that it placed her at an acute risk:

*“I was actually willing to go to medication for the first time ever because I was so bad. I was like okay I might go on an antidepressant(...)That one time I went to A&E was the only time I was actually resorting--- for me to actually accept that okay I might actually take medication I was in a very bad place.” - (Lana)*

To Lana, taking medication was only conceivable when she had to make use of emergency services, denoting it as an option that is ‘resorted’ to at a time of utmost severity. Thus, for all these adolescents, medication was viewed as a resource that can be drawn on when depression was particularly severe and chronic; when they were feeling particularly at their worst and no longer able to manage their depression.

However, for some adolescents, medication featured as a resource only to be drawn on when other options have been exhausted. For example, Charlotte mentioned that medication was only warranted at a time when other treatments have failed, and it was the last option available:

*“I thought try therapy cause then you see if that works and if it does it might help me long-term and if that didn’t work I could always resort to medication”- (Charlotte)*

However, other options were not always described as accessible because of depression severity. For example, Leila described experiencing such chronic low mood and feelings of helplessness that she was unable to manage on her own or engage with help from professionals, at which point she was willing to try medication despite feeling uncertain:

*“I mean I think it was just because I was so low that I couldn’t like benefit from therapy. I was too upset to even like put anything into action or help myself.” - (Leila)*

Medication seemed to be a necessary precursor to being able to access other forms of help. Therefore, these adolescents described medication as the next step when depression felt overpowering and/or nonresponsive to other forms of help. As such, the perceived need for medication acted as a point of reference for adolescents whereby they could gauge not only how severe their depression was, but also how well they were able to manage it.

### Theme 3: ‘A support, not a solution’

The majority of adolescents shared the belief that medication can play a beneficial role whilst overcoming depression. However, its role was deemed to be facilitative, whereby adolescents described medication as a form of additional rather than a solution in its own right. This theme applied to those who had and had not taken medication.

Adolescents who had taken medication described it as playing a key role in their recovery during a time of stagnation, by helping them engage in treatment, regain functioning, and resume pleasurable activities they had stopped due to depression. However, it was seen as offering something additional alongside therapy rather than a standalone treatment—described by Leila as *“extra help from something else”* and by Steven as *“something to lift mood”.* Similarly, Mathilda saw medication as having a supportive role in recovery, whereby it stabilized mood so that it felt manageable.:

*“Therapy was really good, and it helped and probably medication as well, but that was more about the lack of safety blanket until I was feeling better. But I think the main thing was the therapy…just different methods for coping (...) I knew that if things did go downhill again I could just go back on medication I suppose.” -(Mathilda)*

For Mathilda, medication served as a temporary extra component and an option that she could fall back on if needed—particularly at a point of relapse. Further, therapy and medication were seen here as distinct ways of coping, although both were described as contributing to recovery. Similarly, Kayleigh stated that medication allowed her to better engage in therapy and experience a sense of hope despite it initially being a difficult decision:

*“I was getting better but I needed a final push (...)it was a tough decision cause I didn’t like taking tablets but I think looking back now it was the right decision to go on it (...)I started crawling out of the hole but then once the medication kicked in it was just like I can actually see the light at the end.” - (Kayleigh)*

For Kayleigh, combining medication with therapy was described as a turning point in her treatment, whereby medication offered a different kind of support that she experienced as essential to her recovery.

Similarly, to those who did take medication, those who did not talked about how medication might help ease the burden of depression. However, they expressed a desire to deal with underlying issues, which contributed to their decision to refuse medication:

*“Medication doesn’t solve the problem it just helps you deal with it a bit more cause at the end of the day you’re still gonna have to deal with it yourself. Even if medication takes a bit off the edge…it just kind of covers the problem” - (Lana)*

As such, they tended to see medication as a temporary way of managing their depression and preferred to enhance their own ability to cope, describing this as more sustainable in the long-term:

*“The medications not always gonna be there so I would just come to terms with myself and stuff.” - (Callum)*

These adolescents also expressed a preference for therapy: *“if you just take a tablet, you can’t just expect to get better straight away. It’s better to talk things out” (Priya),* appearing to believe that it was a more long-term solution than medication. Natasha, who was offered medication, but declined, cited this preference as her reason. Moreover, she expressed a belief that medication could not help her resolve her difficulties, despite acknowledging that it might temporarily help her manage her symptoms:

*“If I got straight away offered medication without dealing with the real issues, I would have just refused(...)Medication would make me feel okay, but it wouldn’t deal with my deeper demons. I think if we just give someone medication and not give them opportunity to try and explore and work with their issues within themselves…It’s just gonna deal with the problem there and then but it’s not going to help in the long-term” - (Natasha)*

For Natasha, her belief that her depression was linked to relational difficulties seemed to be a key reason for her refusal of medication and preference for therapy:

*“Me having therapy has helped my life in so much ways, it’s gonna help my future in the long run. But if you gave me medication, I would still be battling with my past, my Dad’s relation, and I would just be kind of a neutral person, coping with pills.” - (Natasha)*

Here, medication was described as a temporary method of coping with low mood, whereby the perceived cause of depression remains unaddressed.

Thus, adolescents appeared to denote medication as having the specific role of alleviating low mood, while therapy was seen as addressing underlying issues. Although all adolescents assigned some value to medication, whether they took it seemed to be related to what they felt was the most pertinent to overcoming their depression. Specifically, adolescents who took medication described experiencing low mood as overpowering and debilitating, and thus felt that medication was a necessary step to take to enable them to address underlying issues in therapy. Whereas, adolescents who did not take medication, felt they were functioning well enough and could manage their depression without medication, preferring to deal with underlying issues in the first instance. Still, they did see medication as being available to help them cope with low mood if necessary.

### Theme 4: ‘An ongoing process of trial and error’

This theme relates solely to adolescents who had taken medication. To these adolescents, taking medication was not a straightforward process, rather, it was an ongoing, recursive process of trial and error during which they struggled to find the right type, dosage, frequency, and length of treatment. For example,

*“I was taking anti-depressants for six, seven months, and then eventually I got to a point, before the psychiatrist said I could… I came off medication, but in October I went back onto medication” – (Ada).*

Adolescents also described their struggle to cope with side effects, sometimes having to experiment with different types of medication and understand how they affected their bodies:

*“we went through different antidepressants then I chose one I haven’t taken before (…) Dunno I hope they help but with every anti-depressant there’s a different side-effect…so interesting to see what the side-effects of these are (...)Oh [medication 1]…I just slept constantly(...) and the other ones I just felt completely numb that’s why I stopped taking them…”– (Steven)*

Adolescents also described the difficulty of gaging whether to stop taking medication, which induced a level of anxiety about withdrawal effects. Talia recalled how her decision to suddenly stop medication caused concern from her parents and professionals, resulting in a period of careful monitoring afterwards:

*“I was on fluoxetine twice. I was on it for like a year and then it stopped working and then we tried it again, like a year or so later and it didn’t have the same effect as when I started the first time so we stopped that (...) then I was on citalopram and then spring this year I was like I don’t wanna take these anymore so I stopped (...)Mum and dad were worried, they’re like ‘you ‘can’t just stop taking your medications”- (Talia)*

Thus, for these adolescents, the experience of taking medication was more than merely receiving a prescription and taking it. Rather, it was an ongoing process whereby adolescents wrestled with tough questions relating to the meaning and potential consequences of medication use.

## Discussion

The current study explored the meanings that adolescents attached to SSRI medication within their narratives of overcoming depression. Four main themes were identified: ‘a perceived threat to autonomy’, ‘a sign of severity’, ‘a support, not a solution’, and ‘an ongoing process of trial and error’.

The first theme, ‘a perceived threat to autonomy’ is consistent with previous studies that have shown that adolescents who are resistant to medication tend to describe taking it as inconsistent with being ‘autonomous’ [10, 12, 15]. Adolescence is recognised as a key period for identity formation whereby individuals strive for autonomy, thus adolescents may view medication as a further impingement on their identity as autonomous individuals [24]. Moreover, some adolescents in this study associated antidepressant use with being ‘dependent’ and referred to medication as ‘added chemicals’ that are an undesirable means of obtaining happiness, expressing their wish to do so by their own means. This could indicate some anxiety about what medication does to their body, but also that medication use could be perceived as a loss of control over their bodies, especially at a time when they may be experiencing significant biological changes [25]. Furthermore, this theme was salient for adolescents who had refused to take medication and those who accepted it then decided to stop. Thus, this could have implications for whether adolescents seek or accept medication as part of treatment and whether they adhere to it. Even if antidepressant treatment is effective, it has limited value if adolescents do not seek it and adhere to it [1]. A systematic review found that adolescents’ positive beliefs about their treatment was associated with improved adherence [26].

The second theme, ‘a sign of severity’ showed that medication was deemed necessary when depression was perceived and experienced as severe. This aligns with the NICE recommendations’ stepped care model, which indicates that antidepressants should only be prescribed for adolescents presenting with moderate-to-severe depression [6]. However, all adolescents in this study had a diagnosis of moderate-to-severe depression, indicating there may be qualitative differences in ‘severity’. Adolescents captured this difference, describing a level of ‘severity’ that warrants antidepressant use, associated with the experience of depression as too overwhelming and debilitating, having extremely low mood, and feeling they could no longer manage on their own. These descriptions have been reported by other adolescents in previous qualitative research [27, 28], where this ‘overwhelming’ quality to depression was also prominent.

Moreover, adolescents’ descriptions of feeling too low to benefit from therapy, feeling trapped by depression, and being at a dangerous low point are consistent with previous findings that suggest adolescents are prescribed antidepressants as a first line treatment when they present with severe depression or are too reluctant to initially engage with therapy [29]. Therefore, introducing antidepressants at an earlier stage in treatment may be beneficial to some adolescents. This is supported by the latest NICE guidelines which suggest antidepressants as a first line treatment in combination with psychological therapy or on their own if the adolescents do not engage with psychological therapy [6].

Adolescents’ understanding of the need to take medication also seemed related to how they perceived their ability to cope. This ties in with the first theme, which suggests medication is perceived as a sign of being incapable of managing depression via their self-determination. These two themes may indicate that taking a daily pill to cope with depression could be perceived by adolescents as a concrete statement that they are at a particularly low point, unable to manage without it. As such, these themes echo the findings of other studies in which adolescents described psychiatric medication as a marker of their ‘defectiveness’ and or a sign that they are ‘not autonomous’ [10, 12, 15]. Underlying beliefs of being ‘defective’ and ‘dependent’ are also characteristic of depression more generally [30]. When these meanings are associated with medication, it is possible that the suggestion of medication may trigger, or heighten, these beliefs. Given that adolescence is recognized as a developmental stage in which issues of identity and autonomy are particularly salient, these beliefs may impact adolescents’ views of themselves and have longer term consequences if not addressed [14]. This highlights the need for further research and careful consideration when prescribing antidepressant medication, specifically in relation to how adolescents’ personal meanings might interact with relevant developmental issues.

The third theme, ‘a support, not a solution’ is consistent with previous qualitative research with adults where antidepressants were described as helpful to cope with and improve low mood, but only a partial or temporary fix [31, 32] or as not addressing social and psychological issues that were perceived as causes of depression [33]. However, the current findings indicate that while adolescents seem to assign SSRIs and psychological therapy distinct roles in treatment, they do not discount medication as an option. Whether and when adolescents believe SSRIs to be beneficial to their treatment could be related to whether they believe alleviating symptoms or addressing other psychological or social issues was most pertinent to their recovery. This also links to the previous theme, where adolescents felt that medication could play a beneficial role in treatment when depressive symptoms did not allow them to address underlying issues in therapy. Subsequently, the value adolescents place on the role of antidepressants in their treatment could be related to their beliefs about their depression. Although both adolescents who had and had not taken medication seemed to share the view that the act of taking antidepressants implies that they are not autonomous or that their depression is particularly severe, they simultaneously maintained that medication can play a facilitative role in treatment. These results are consistent with those reported in studies with adults, which show that they to struggle with ambivalent views about antidepressant medication [32, 34, 35]. These simultaneous beliefs could create a dilemma for adolescents who are deciding whether to take medication for the first time, or for those who are taking medication, whether to stop.

The fourth theme, ‘an ongoing process of trial and error’ highlights that taking antidepressants can be a difficult process marked by a continuous struggle to enhance benefits and reduce adverse effects. This supports NICE guidelines’ emphasis on close and continuous monitoring of adolescents taking antidepressants [6]. However, this theme highlights how frustrating and disillusioning this process can be, potentially heightening feelings of hopelessness, which are associated with depression [36]. Thus, more holistic support is required in addition to routine monitoring, holding implications for both treatment effectiveness and adherence. Additionally, it emphasizes that adolescents may have concerns about when to stop their medication, what it means to function without it, or difficult withdrawals—a significant issue for adolescents discontinuing antidepressants [37]. Qualitative studies have found that adults may choose to remain on antidepressants despite perceiving that they no longer need them due to fears of withdrawal, relapse, or lack of support [38, 39].

Together, these four themes highlight how antidepressant treatment can hold complex meanings for adolescents throughout their experience of overcoming depression and may have implications for their views of themselves and the way they conceptualize their depression. This aligns with adult literature showing that experiences with antidepressant use are complex and dynamic, characterized by a temporal process of ‘decision-making’ and ‘meaning-making’ where treatment decisions are interlinked with the emergence of new self-concepts [32, 40].

### Clinical implications

This study holds implications regarding antidepressant treatment with adolescents. Particularly, it re-emphasizes that practitioners should involve adolescents in the discussion about risks and benefits of SSRIs and provide them with the latest information to help them make informed choices and that antidepressant treatment should be monitored continuously [6]. These findings also emphasize that in addition to addressing issues of symptom control, physical tolerability, adherence, and side effects, clinicians should continuously and actively elicit, address and reflect on the impact of medication on adolescents’ perceptions of themselves, their depression, and their quality of life with consideration to their developmental context. Further, this study supports the need for better implementing models of shared decision making (SDM) and involving adolescents as active partners their antidepressant treatment advocated by others [41–46].

### Strengths and limitations

The current study elucidated meanings grounded in adolescents’ perspectives on antidepressant use, which have scarcely been explored. Further, it captured specific nuances of meaning and potential commonalities in adolescents’ views and experiences. However, as all the adolescents sought out, engaged with, and completed psychological therapy by the time of interview, these findings may not be representative of those who do not seek help, drop out of treatment, or do not engage with therapy and opt for solely taking antidepressant medication. Adolescents were also interviewed at the final time point of the IMPACT-ME study and the interviews were selected only if they spoke about their experience or feelings about medication—which was not the interviews’ primary focus. Therefore, these findings may only be representative of adolescents who had strong opinions on medication and were invested in expressing their views and experiences and cannot be extrapolated to all depressed adolescents. Additionally, meanings were derived from adolescents’ retrospective accounts. Thus, it is difficult to discern whether and how these meanings may have shifted over time from initial discussions.

## Conclusions

Overall, the current study highlights the value of bringing adolescents’ voices into the broader debate on the use of antidepressants in their age group and in the development of future guidelines. The findings showed how adolescents’ may attribute highly personal meanings to antidepressant use, which hold implications for their view of themselves and their depression, and in turn, may impact on their treatment decision, their adherence, their response to medication if it is taken. Hence, the current study also emphasizes the importance of collaboratively exploring these meanings with adolescents throughout the treatment process in order to enhance their treatment experience.

The findings of this study also echo previous literature advocating a shift from questioning whether antidepressants work to questioning whom they work for [44, 47]. They highlight the value of incorporating qualitative research from an adolescent-centered perspective into future NICE guidelines as is already the case in relation to guidelines on ADHD medication use [48]. Future research should explore adolescents’ perspectives of SSRIs at different stages of treatment, which may provide insight into how to better support adolescents during the course of their treatment. Over time it may be possible to create a valuable body of literature that can be referred to when making life-changing decisions about whether to initiate, continue, or discontinue antidepressant treatment with adolescents.

## Abbreviations

BPI: Brief psychosocial intervention
CAMHS: Child and adolescence mental health services (CAMHS)
CBT: Cognitive behavioral therapy
IMPACT: Improving moods with psychoanalytic and cognitive therapies
IMPACT-ME: Impact my experience
MDD: Major depressive disorder
NICE: National Institute for Health Care and Excellence
SSRIs: Selective serotonin reuptake inhibitors
STPP: Short-term psychodynamic psychotherapy
TA: Thematic analysis
